# Interaction of *Bdellovibrio bacteriovorus* with Gram-Negative and Gram-Positive Bacteria in Dual Species and Polymicrobial Communities

**DOI:** 10.3390/microorganisms10040793

**Published:** 2022-04-09

**Authors:** Monique Waso-Reyneke, Sehaam Khan, Wesaal Khan

**Affiliations:** 1 Faculty of Health Sciences, University of Johannesburg, P.O. Box 17011, Doornfontein 2028, South Africa; 221029180@student.uj.ac.za (M.W.-R.); skhan@uj.ac.za (S.K.); 2 Department of Microbiology, Faculty of Science, Stellenbosch University, Private Bag X1, Stellenbosch 7602, South Africa

**Keywords:** *Bdellovibrio bacteriovorus*, predation efficiency, polymicrobial communities, mixed microbial communities, predatory bacteria, prey selection

## Abstract

The interaction of *Bdellovibrio bacteriovorus* PF13 with mixed bacterial communities, consisting of Gram-negative (*Pseudomonas fluorescens* and *Klebsiella pneumoniae*) and Gram-positive (*Staphylococcus aureus* and *Enterococcus faecium*) bacteria, was investigated to determine if this wild-type predator preferentially preys on certain bacteria and whether the presence of Gram-positive organisms influences its predation efficiency. In co-culture with *P. fluorescens* and *K.* *pneumoniae*, the cell counts (PFU/mL) of PF13 increased by 5.79 and 5.17 logs (48 h), respectively, while in the dual species assay (*P. fluorescens*, *K. pneumoniae* and PF13), the cell counts of PF13 increased by 1.95 logs (24 h). Using ethidium monoazide bromide quantitative polymerase chain reaction (EMA-qPCR), the concentration of PF13 increased by 1.25 to 3.62 logs in the co-culture experiments, by 1.41 to 5.05 logs in dual species cultures and by 2.65 logs in a polymicrobial culture. However, PF13 preferentially preyed on *K. pneumoniae* in the dual species and polymicrobial cultures, highlighting that the presence of Gram-positive bacteria did not affect the predation efficiency of PF13. This is significant as it implies that the predator can be applied in mixed microbial communities to target Gram-negative pathogens which may pose a health risk to patients, consumers or for the treatment of contaminated water.

## 1. Introduction

The predatory bacteria *Bdellovibrio bacteriovorus* (*B. bacteriovorus*), *Peredibacter*, *Bacteriovorax*, *Halobacteriovorax* and *Micavibrio*, are collectively referred to as the *Bdellovibrio*-and-Like-Organisms (BALOs) [1]. Apart from the epibiotic predators (which leach the cellular contents from their attacked prey cells) such as *Micavibrio* and *Bdellovibrio exovorus*, the BALOs are generally characterised by a biphasic life cycle where they invade (periplasmic predators) and eventually lyse the prey bacteria. As a result of their predation activity, it has been suggested that the BALOs play a crucial ecological role by controlling bacterial numbers, shaping microbial communities, and contributing to biogeochemical cycling [1,2].

It is well established that this group of predators predominantly prey on Gram-negative bacteria; however, it is not clear why they prefer this group of organisms or how they identify prey, especially in mixed microbial communities [1]. Negus et al. [3] highlighted that a multifactorial process is likely used by the BALOs to identify and attack prey. Specifically, locomotion (high speed swimming and gliding motility), chemotaxis and attachment to prey via type IV pili, are some of the factors that have been identified to play a role in predation [3]. Additionally, Schwudke et al. [4] reported that *B. bacteriovorus* has a unique, uncharged lipid A molecule in its outer membrane. These unique lipid A molecules are thought to contribute to self-recognition and may aid *B. bacteriovorus* in identifying prey, allow close contact between *B. bacteriovorus* and other bacteria, and may confer resistance to cationic antibiotics [4]. Williams and Chen [1] also noted that the environment a predatory bacterium is isolated from (specifically the temperature and salinity of that environment) and the prey bacteria present in that environment, may determine the prey range as well as the efficiency with which predatory bacteria prey on other bacteria. Similarly, Saralegui et al. [5] highlighted that predator–prey interactions may be specific to the predator–prey strain combinations and that these interactions may be influenced by the origin and not necessarily the genetic composition of the isolates or even their antimicrobial profiles; however, much still remains to be elucidated regarding prey preference and how *B. bacteriovorus* selects its prey.

The most widely studied predatory bacteria and the BALO that will be focused on in this study is *B. bacteriovorus*. This predator exhibits a relatively wide prey range, which includes various Gram-negative bacteria such as *Escherichia coli* (*E. coli*), *Pseudomonas* spp., *Klebsiella* spp. and *Enterobacter* spp. [6]. A few studies have also indicated that *B. bacteriovorus* can interact with Gram-positive bacteria such as *Staphylococcus aureus* (*S. aureus*) in order to survive [7,8,9,10]. The predation efficiency and the prey range of *B. bacteriovorus* has primarily been investigated in laboratory co-culture experiments (*B. bacteriovorus* exposed to one strain of prey bacteria), with the *B. bacteriovorus* type strains HD100 and 109J generally employed as the primary predators [1]. However, limited research has been conducted on the effect of *B. bacteriovorus* on mixed microbial communities and in turn, the effect of mixed microbial communities on the predation efficiency of *B. bacteriovorus* [6,11,12,13,14].

Hobley et al. [12] investigated the interaction of *B. bacteriovorus* HD100 with a mixed culture containing a predation sensitive organism (*E. coli*; Gram-negative) and predation resistant organism [*Bacillus subtilis* (*B. subtilis*); Gram-positive]. Results indicated that while *B. bacteriovorus* was able to reduce the concentration of *E. coli* from ~10^8^ cells/mL to ~10^5^ cells/mL in the presence and absence of *B. subtilis*, the concentration of *E. coli* was reduced at a slower rate when *B. subtilis* was present. The authors also indicated that the predation rate of *B. bacteriovorus* was reduced because the predator would collide with the *B. subtilis* cells, remain attached for 1 to 5 min and then detach to search for susceptible prey. In contrast, Rogosky et al. [13] investigated the interaction of *B. bacteriovorus* 109J with mixed cultures containing two predation sensitive bacteria. The authors investigated the following combinations of prey (1:1 ratio): *Serratia marcescens* (*S. marcescens*) and *Pantoea agglomerans* (*P. agglomerans*); *E. coli* and *P. agglomerans*; *Enterobacter aerogenes* (*E. aerogenes*) and *S. marcescens*; *E. coli* and *S. marcescens; Salmonella enterica* (*S. enterica*) and *S. marcescens*; and *Erwinia carotovora* (*E. carotovora*) and *S. marcescens*. From the mixed culture data, the authors concluded that *B. bacteriovorus* 109J preferentially preyed on *P. agglomerans* and *S. marcescens* and when these two prey were combined, *P. agglomerans* was preferred over *S. marcescens*. It was thus concluded that when *B. bacteriovorus* 109J is exposed to two predation sensitive bacteria available in the same abundance, it may select and preferentially attack one bacterium. However, given the application of *B. bacteriovorus* in the food, water and medical fields [15,16], it is important to investigate the predation strategy of this bacterium in a mixed microbial community, where a higher diversity (more than two species or strains) of prey and non-prey bacteria may be present.

Based on the theory that prey preference and selection may be influenced by the environment *Bdellovibrio* strains are isolated from, we hypothesise that wild-type bdellovibrios, that exist in mixed microbial communities, selectively prey on bacteria they are frequently exposed to. Furthermore, as limited research has been conducted on the application and predation strategy of wild-type *B. bacteriovorus* strains, the aim of this study was to investigate the interaction of a wild-type *B. bacteriovorus* strain (*B. bacteriovorus* PF13) with mixed bacterial communities consisting of two Gram-negative (predation sensitive) strains, one Gram-negative and one Gram-positive strain, as well as two Gram-negative and two Gram-positive strains, in order to determine if the presence of multiple prey strains has an effect on the predation efficiency of wild-type bdellovibrios. *Bdellovibrio bacteriovorus* PF13 was previously isolated from wastewater using *Pseudomonas fluorescens* (*P. fluorescens*) as prey [17]. This predator has been found to prey on the Gram-negative organisms *Pseudomonas aeruginosa* (*P. aeruginosa*), *P. fluorescens* and *Klebsiella pneumoniae* (*K. pneumoniae*) and it grows in the presence of the Gram-positive bacteria, *Enterococcus faecium* (*E. faecium*) and *S. aureus* [17].

## 2. Materials and Methods

### 2.1. Bacterial Strains and Cultivation

The predatory bacterium *B. bacteriovorus* PF13 was stored as plaques on diluted nutrient broth (DNB) double-layer agar overlays with *P. fluorescens* ATCC 13525 as prey [6,18]. To apply *B. bacteriovorus* PF13 in the co-culture, dual species and polymicrobial assays, a stock lysate was prepared in DNB with *P. fluorescens* ATCC 13525 as prey as described by Waso et al. [18]

**Figure 1:**
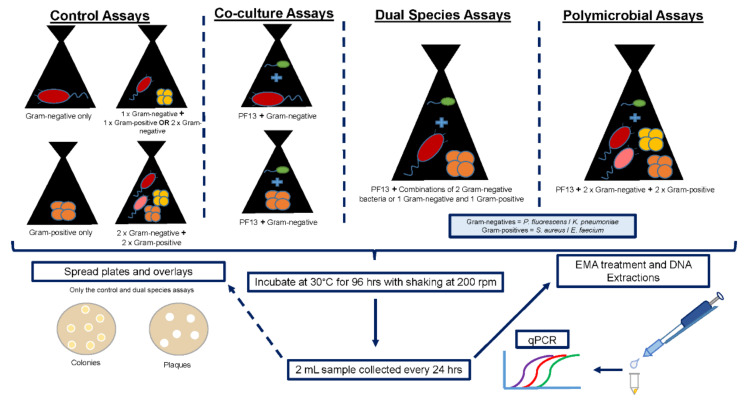
Summary of the control, co-culture, dual species and polymicrobial assays and the processing of the samples for culture- and molecular-based assays.

The prey strains utilised for the experimental assays were *P. fluorescens* ATCC 13525, *K. pneumoniae* ATCC 333305, *S. aureus* ATCC 25925 and an *E. faecium* clinical isolate. These strains were obtained from the Water Resource Laboratory culture collection at Stellenbosch University and were streaked onto Luria Bertani (LB) agar (Biolab, Merck, South Africa) from glycerol stocks stored at −80 °C. The plates were incubated at 30 °C for 24 to 48 h. A single colony of each strain was subsequently inoculated into 200 mL LB broth (Biolab) and incubated for 18 to 24 h at 30 °C with shaking at 120 rpm. The cells were harvested by centrifugation at 8000 rpm for 20 min, whereafter the cells were washed and resuspended in DNB to an optical density at 600 nm (OD_600_) of 1.00. These prey cell suspensions were then used to inoculate the various experimental assays (Figure 1).

### 2.2. Co-Culture Assays

To assess the interaction between *B. bacteriovorus* PF13 and the individual Gram-negative and Gram-positive organisms, co-culture assays were conducted. *Bdellovibrio bacteriovorus* PF13 was thus combined with one Gram-negative or one Gram-positive organism in DNB.

To conduct these experiments, 80 mL of DNB was inoculated with 10 mL *B. bacteriovorus* PF13 stock lysate (OD_600_ < 0.2) and 10 mL of the Gram-negative bacteria (OD_600_ = 1.00) or 10 mL of the Gram-positive bacteria (OD_600_ = 1.00) in 250 mL Erlenmeyer flasks (Figure 1). These flasks were incubated at 30 °C for 96 h with shaking at 120 rpm. Control cultures consisted of 90 mL of DNB inoculated with 10 mL of the Gram-negative bacteria (OD_600_ = 1.00) or 10 mL of the Gram-positive bacteria (OD_600_ = 1.00) in 250 mL Erlenmeyer flasks (Figure 1). All experiments were conducted in duplicate.

Samples (2 mL) were collected from each of the experimental and control flasks every 24 h with the respective prey cells enumerated using colony forming units (CFU) and the predator cells enumerated using plaque forming units (PFU), as described in the Section 2.5 (Figure 1). The samples were also processed using ethidium monoazide bromide quantitative polymerase chain reaction analysis (EMA-qPCR) as outlined in the Section 2.6 (Figure 1).

### 2.3. Dual Species Assays

*Bdellovibrio bacteriovorus* PF13 was exposed to dual species cultures (two bacterial strains exposed to the predator) to determine if PF13 preferentially preys on certain strains or if the presence of Gram-positive bacteria influences the predation efficiency of PF13. The bacteria were thus combined as follows: *P. fluorescens* and *K. pneumoniae*; *P. fluorescens* and *S. aureus*; *P. fluorescens* and *E. faecium*; *K. pneumoniae* and *S. aureus*; and *K. pneumoniae* and *E. faecium* (Figure 1).

To conduct these experiments, 80 mL of DNB was inoculated with 10 mL *B. bacteriovorus* PF13 stock lysate and 5 mL of each Gram-negative strain (OD_600_ = 1.00) or 5 mL of the Gram-positive bacteria (OD_600_ = 1.00) and 5 mL of the Gram-negative bacteria (OD_600_ = 1.00) in 250 mL Erlenmeyer flasks (Figure 1). Control cultures consisted of 90 mL of DNB inoculated with 5 mL of each Gram-negative strain (OD_600_ = 1.00) or 5 mL of the Gram-positive bacteria (OD_600_ = 1.00) and 5 mL of the Gram-negative bacteria (OD_600_ = 1.00) (Figure 1). These flasks were incubated at 30°C for 96 h with shaking at 120 rpm. All experiments were conducted in duplicate.

Samples (2 mL) were collected and analysed as described in Section 2.2, Section 2.5 and Section 2.6.

### 2.4. Polymicrobial Assay

To determine if *B. bacteriovorus* PF13 selects prey or randomly attacks susceptible prey in a mixed bacterial community consisting of more than two bacterial strains, *B. bacteriovorus* PF13 was exposed to a polymicrobial culture consisting of two Gram-negative and two Gram-positive bacterial strains. Eighty millilitre (80 mL) DNB flasks were inoculated with 2.5 mL of *P. fluorescens*, *K. pneumoniae*, *S. aureus* and *E. faecium* cells suspended in DNB at an OD_600_ of 1.00 (Figure 1). Each experimental flask was also inoculated with 10 mL *B. bacteriovorus* PF13 stock lysate. For the controls, 90 mL of DNB was inoculated with only 2.5 mL of *P. fluorescens*, *K. pneumoniae*, *S. aureus* and *E. faecium* cells suspended in DNB at an OD_600_ of 1.00 (Figure 1). These flasks were incubated at 30 °C for 96 h with shaking at 120 rpm and the experiments were conducted in duplicate. Samples (2 mL) were collected from each of the experimental and control flasks every 24 h and these samples were processed using EMA-qPCR as outlined in Section 2.6 (Figure 1).

### 2.5. Culture-Based Analysis

Selective media were used to enumerate the CFU of the respective bacteria utilised in the co-culture and the dual species assays as follows: Cetrimide Agar Base (Biolab, Merck, South Africa) for *P. fluorescens*; HiCrome^™^ *Klebsiella* Selective Agar Base (Sigma-Aldrich, South Africa) for *K. pneumoniae*; Mannitol Salt agar for *S. aureus* (Biolab, Merck, South Africa); and Slanetz and Bartley agar (Oxoid, Thermo Fisher Scientific, Cape Town South Africa) for *E. faecium*. The media were prepared as described in the manufacturers’ instructions. The prey cell counts were enumerated by preparing serial dilutions (10^−2^ to 10^−8^) of the samples in saline (0.85% NaCl) collected every 24 h (from 0 to 96 h) from the respective flasks. The dilutions were subsequently spread plated onto the relevant selective media agar plates in triplicate. The plates were incubated at 30 °C for 18 to 24 h, whereafter the CFU were recorded.

To enumerate *B. bacteriovorus* PF13, serial dilutions (10^−2^ to 10^−8^) of the samples collected from the respective flasks were prepared, whereafter double-layer agar overlays as described by Yu et al. [19] were completed. For the co-culture assays where *B. bacteriovorus* was exposed to a single bacterial strain, 500 µL of each dilution and 500 µL of fresh bacterial cells (OD_600_ = 1.00) was added to 5 mL soft-top agar to prepare the overlays. For the experimental samples where *B. bacteriovorus* was exposed to two organisms, 500 µL of each dilution and 250 µL of each bacterial strain (OD_600_ = 1.00) was added to 5 mL soft-top agar to prepare the overlays. The plates were incubated at 30 °C for up to 7 days and the PFU on each plate were recorded.

### 2.6. Molecular Analysis

The samples collected from the controls, the co-culture assays, the dual species assays and the polymicrobial assays were processed using EMA-qPCR analysis, to determine the concentration of potentially viable Gram-negative, Gram-positive and predatory bacteria in the respective samples. Briefly, 500 µL of each 2 mL sample was treated with EMA as described by Reyneke et al. [20] and the DNA was extracted from these samples using the Zymo Research Quick DNA Fecal/Soil Microbe MiniPrep™ kit as per the manufacturer’s instructions. The gene copies (GC) of the respective Gram-negative and Gram-positive bacteria and *B. bacteriovorus* PF13 were determined using the primers and cycling parameters as outlined in the Supplementary Materials Table S1. The qPCR components were combined and the standard curves for each qPCR assay were generated as outlined in Waso et al. [17].

### 2.7. Statistical Analysis

The qPCR performance parameters were determined using the Roche LightCycler^®^96 Software version 1.1 (Supplementary Materials Table S2). The qPCR data were processed and the GC/µL were converted to GC/mL using Microsoft Excel 2016 as described in Waso et al. [17] Analysis of Variance (ANOVA) was applied to determine if the CFU, PFU or GC changed significantly in the experiments. Significance was observed as *p* < 0.05.

As a measure of predation efficiency, the percentage bacterial cells remaining after 96 h of exposure to PF13 relative to the cells remaining in the control cultures (DNB containing only the prey bacteria) was calculated as described by Rogosky et al. [13] Briefly, the GC detected using the EMA-qPCR assays were converted to cell equivalents (CFU/mL) by dividing the number of GCs detected with the EMA-qPCR assays with the number of the target genes present in the genome of the target bacteria (calculations not shown). The percentage presumably viable cells after 96 h was then calculated by dividing the cells detected at 96 h in a predated culture (PF13 with a single prey organism; PF13 with two organisms; and PF13 with all four organisms) by the cells detected at 0 h. Similarly, the cells detected at 96 h in the culture not exposed to PF13 (cultures containing only one Gram-negative or Gram-positive; cultures containing two organisms; and, cultures containing all four organisms) was divided by the cells detected at 0 h. The predation efficiency of PF13 was subsequently calculated by dividing the percentage of presumably viable cells detected in the culture exposed to PF13 by the percentage cells remaining in the absence of PF13 [12].

## 3. Results and Discussion

Most studies have investigated the physiology and life cycle of *Bdellovibrio* using *E. coli* as the model prey bacteria and apart from prey susceptibility testing used for *Bdellovibrio* strain typing, limited research has been conducted on the predation efficiency and interaction of *Bdellovibrio* with other prey strains [13]. Additionally, studies generally combine *Bdellovibrio* with individual bacterial strains and subsequently enumerate the bacterial cells to determine survival, or measure *Bdellovibrio* PFU to monitor predator growth [13]. There is thus a lack of research investigating: (i) the efficiency of *Bdellovibrio* when exposed to mixed bacterial cultures; (ii) the prey ranges and predation efficiencies of wild-type bdellovibrios; (iii) the predation efficiency in the presence of multiple susceptible and non-susceptible prey; and (iv) the predation efficiency of bdellovibrios in their natural environments versus in vitro studies. Therefore, to fully understand the potential applications of these predators, there is a need to address these gaps in predatory bacteria research. The aim of this study was thus to investigate the interaction and predation efficiency of a wild-type bdellovibrio strain, *B. bacteriovorus* PF13, exposed to mixed bacterial communities comprised of predation sensitive and non-sensitive bacteria.

*Bdellovibrio bacteriovorus* PF13 was subsequently exposed to a combination of two Gram-negative bacteria (*P. fluorescens* and *K. pneumoniae*) or one Gram-positive (*S. aureus* or *E. faecium*) and one Gram-negative (*P. fluorescens* or *K. pneumoniae*) bacterium in the dual species assays. For comparison with these dual species assays, *B. bacteriovorus* PF13 was cultured with one Gram-negative or one Gram-positive bacterium in co-culture experiments. The trends observed in the co-cultures of *B. bacteriovorus* PF13 with *P. fluorescens*, *K. pneumoniae*, *S. aureus* or *E. faecium,* respectively, will be described first, followed by the trends observed in the dual species assays and lastly the results obtained for the polymicrobial assay (all test strains combined with *B. bacteriovorus* PF13) will be presented. All of the data for the control assays (Figure 1) are presented in Supplementary Materials Table S3.

### 3.1. Co-Culture of B. bacteriovorus PF13 with P. fluorescens, K. pneumonia, S. aureus or E. faecium

When *B. bacteriovorus* PF13 was exposed to *P. fluorescens* in the co-culture experiments, the concentration of PF13 increased by 5.79 logs to a maximum of 1.30 × 10^9^ PFU/mL (at 48 h), whereafter the counts reduced (Table 1). The EMA-qPCR analysis indicated that the concentration of PF13 increased by 3.62 logs after 96 h (Table 1). Correspondingly, the concentration of *P. fluorescens* was reduced by 2.91 logs (Table 2A), with the EMA-qPCR analysis indicating that the concentration of *P. fluorescens* was reduced by 1.58 logs (Table 2A).

When *B. bacteriovorus* PF13 was exposed to *K. pneumoniae* in the co-culture experiments, the concentration of PF13 increased by 5.17 logs to a maximum of 2.10 × 10^9^ PFU/mL (at 48 h), whereafter the counts reduced (Table 1). The EMA-qPCR analysis indicated that the concentration of PF13 increased by 2.82 logs after 96 h (Table 1). Correspondingly, the cell counts of *K. pneumoniae* were reduced by 3.73 logs (Table 2B), with the EMA-qPCR analysis indicating that the concentration of *K. pneumoniae* was reduced by 1.91 logs (Table 2B).

It should be noted that the cell counts (PFU/mL) for *B. bacteriovorus* PF13 could not be determined when Gram-positive bacteria were used, or when PF13 was exposed to a mixture of Gram-positive and Gram-negative prey (dual species assays), as PF13 did not form visible plaques on these overlays. This could be attributed to the fact that *B. bacteriovorus* is not able to effectively lyse Gram-positive bacteria, and therefore clearing of the cells from the agar, which results in plaque formation, is not observed on the double-layer agar overlays where Gram-positive bacteria are present. Therefore, only the EMA-qPCR analysis for PF13 will be presented for the Gram-positive co-cultures and the dual species samples.

When *B. bacteriovorus* PF13 was co-cultured with *S. aureus*, the EMA-qPCR analysis indicated that the concentration of PF13 increased by 1.25 logs (Table 1). Based on the culturing analysis, the concentration of *S. aureus* was reduced by 1.94 logs (Table 2C), while the EMA-qPCR analysis indicated that the concentration of *S. aureus* was reduced by 0.88 logs (Table 2C). For the co-culturing of *B. bacteriovorus* PF13 with *E. faecium*, the EMA-qPCR analysis indicated that the concentration of PF13 increased by 2.56 logs (Table 1). The concentration of *E. faecium* was then reduced by 1.43 logs (Table 2D) based on the culturing analysis and by 0.78 logs using EMA-qPCR (Table 2D).

*Pseudomonas fluorescens* has been identified as an emerging opportunistic pathogen, which can cause bloodstream, bone, cerebrospinal, eye, lung, sinus, skin, wound and urinary tract infections [21]. In addition, *K. pneumoniae* is considered an important opportunistic pathogen and has been included in the ESKAPE pathogen group (*Enterococcus faecium*, *Staphylococcus aureus*, *Klebsiella pneumoniae*, *Acinetobacter baumannii*, *Pseudomonas aeruginosa* and *Enterobacter* spp.) because of its drug resistance profile [22]. Extensive research has thus focused on the application of *B. bacteriovorus* as a “living antimicrobial” or biocontrol agent in the medical, agricultural and food industries to control *Klebsiella* and *Pseudomonas* spp. [15,16] For example, Saxon et al. [23] showed that *B. bacteriovorus* could reduce the brown blotch lesions, caused by *Pseudomonas tolaasii*, on supermarket mushrooms and potentially aid in preventing food spoilage of commercial mushrooms. Kadouri et al. [24] then showed that *B. bacteriovorus* is effective in killing multidrug-resistant *P. aeruginosa*, *P. putida* and *K. pneumoniae* strains, which may have significant implications for clinical applications. Moreover, Shatzkes et al. [25] showed that *B. bacteriovorus* can reduce the concentration of *K. pneumoniae* in the lungs of rats by more than 3 logs.

Although limited research has been conducted on the interaction of *B. bacteriovorus* with Gram-positive bacteria, Monnappa et al. [8] and Im et al. [9] showed that *B. bacteriovorus* is able to produce lytic enzymes, such as proteases that can degrade biofilms produced by *S. aureus*. Iebba et al. [7] further confirmed a direct interaction between *B. bacteriovorus* and *S. aureus* cells and showed a reduction of *S. aureus* biofilm growth by the predator. A previous study by our research group also showed that *B. bacteriovorus* PF13 may interact with *E. faecium* and *S. aureus* in order to survive, as the concentration of the predator increased in the presence of these Gram-positive bacteria, while reductions in the cell counts and GC of *E. faecium* and *S. aureus* were recorded (although this was influenced by the presence (*E. faecium*) or absence (*S. aureus*) of nutrients in the media) [17]. Similarly, in the current study, the GC and cell counts of *S. aureus* and *E. faecium* were reduced, while the concentration of PF13 increased. These results indicate that *B. bacteriovorus* may exhibit a negative effect on Gram-positive bacteria and as *S. aureus* and *E. faecium* are opportunistic pathogens of humans, which have also been included in the ESKAPE pathogens group [22], given the results obtained in the current study, the interaction of *B. bacteriovorus* with other Gram-positive pathogens that negatively impact the medical, food or agricultural sectors, warrants further research.

Interestingly, our data showed that although the double-layer agar overlays indicated a notable decrease in the plaque counts of PF13 from 48 h to 96 h in the co-culture experiments, the gene copies of PF13 remained relatively high at 10^6^ to 10^7^ GC/mL. This may be attributed to the presence of host-independent bdellovibrios, which may not readily form plaques on the double-layer agar overlays. While this phenomenon was not investigated further in the current study, studies are currently being conducted on PF13 to determine whether this wild-type strain is able to switch to host-independent growth and under which conditions this switch occurs. These results also highlight the value of EMA-qPCR analysis in predator–prey interaction studies, as we were able to confirm that PF13 remains viable for up to 96 h.

### 3.2. Dual Species Interaction of B. bacteriovorus PF13 with Combinations of Gram-Negative and Gram-Positive Bacteria

In the dual species assays, when PF13 was simultaneously cultured with *P. fluorescens* and *K. pneumoniae*, the concentration of PF13 increased by 1.95 logs to a maximum of 5.03 × 10^8^ PFU/mL (at 24 h), whereafter the counts were reduced (Table 1). The EMA-qPCR analysis indicated that the concentration of PF13 increased by 1.41 logs (Table 1). Based on the culturing analysis, the concentration of *P. fluorescens* remained relatively constant with 5.17 × 10^8^ CFU/mL recorded at 0 h and 4.98 × 10^8^ CFU/mL recorded at 96 h (Table 2A), while the EMA-qPCR analysis indicated that the concentration of *P. fluorescens* increased by 0.75 logs (Table 2A). In contrast, the concentration of *K. pneumoniae* was reduced by 3.59 logs based on the culturing analysis (Table 2B), and by 1.50 logs as determined using EMA-qPCR (Table 2B).

In the dual species assays, when PF13 was simultaneously cultured with *P. fluorescens* and *S. aureus*, the concentration of PF13 increased by 5.05 logs. Correspondingly, the concentration of *P. fluorescens* was reduced by 3.01 logs based on the culturing analysis (Table 2A), and by 1.22 logs as determined using EMA-qPCR (Table 2A). The concentration of *S. aureus* was then reduced by 1.45 logs based on the culturing analysis (Table 2C), and by 2.13 logs using EMA-qPCR (Table 2C).

When PF13 was simultaneously cultured with *P. fluorescens* and *E. faecium*, the concentration of PF13 increased by 3.29 logs. The concentration of *P. fluorescens* was reduced by 4.59 logs based on the culturing analysis (Table 2A), and by 1.68 logs as determined using EMA-qPCR (Table 2A). The concentration of *E. faecium* was then reduced by 2.32 logs as determined using the culture-based analysis (Table 2D), and by 0.35 logs using EMA-qPCR (Table 2D).

When PF13 was simultaneously cultured with *K. pneumoniae* and *S. aureus*, the concentration of PF13 increased by 4.21 logs. Correspondingly, the concentration of *K. pneumoniae* was reduced by 4.12 logs using the culture-based analysis (Table 2B), and by 2.57 logs as determined using EMA-qPCR (Table 2B). The concentration of *S. aureus* was reduced by 3.12 logs based on the culturing analysis (Table 2C); however, the EMA-qPCR analysis indicated that there was no significant change in the GC from presumably viable *S. aureus* cells with 2.42 × 10^5^ GC/mL detected at 0 h and 2.00 × 10^5^ GC/mL detected at 96 h (0.083 log reduction) (Table 2C).

In the dual species assays, when PF13 was cultured with *K. pneumoniae* and *E. faecium*, the concentration of PF13 increased by 2.93 logs. Correspondingly, the concentration of *K. pneumoniae* was reduced by 3.98 logs as recorded using the culture-based analysis (Table 2B), and by 1.40 logs as determined using EMA-qPCR (Table 2B). The concentration of *E. faecium* was reduced by 0.96 logs using the culture-based analysis (Table 2D), and by 0.59 logs as indicated using the EMA-qPCR analysis (Table 2D).

Limited studies have investigated the interaction of *B. bacteriovorus* with multiple bacteria simultaneously [6,11,12,13,14]. Rogosky et al. [13] reported that when *B. bacteriovorus* is cultured with two predation sensitive bacteria simultaneously, *B. bacteriovorus* may selectively prey on one strain. Similarly, in the current study when *B. bacteriovorus* PF13 was cultured with *P. fluorescens* and *K. pneumoniae* in the dual species assays, this predator preferentially preyed on *K. pneumoniae* as verified by the reduction in cell counts and GC, while the cells counts for *P. fluorescens* remained relatively constant and the GC increased (albeit not significantly). In contrast, Hobley et al. [12] investigated the interaction of *B. bacteriovorus* with a dual species culture consisting of a Gram-negative (*E. coli*) and a Gram-positive (*B. subtilis*) organism. The predator successfully decreased the *E. coli* concentration by approximately 3 logs in the presence and absence of *B. subtilis*, although the concentration of *E. coli* was reduced at a slower rate when *B. subtilis* was present. The authors concluded that the presence of the Gram-positive bacteria did not hinder predation on the Gram-negative organisms, but it did reduce the speed at which the Gram-negative bacteria are attacked and killed. Similarly, Im et al. [14] indicated that *B. bacteriovorus* HD100 reduced the concentration of *A. baumannii* and *K. pneumoniae* by approximately 4 logs in dual species assays that consisted of *A. baumannii* combined with *S. aureus* or *K. pneumoniae* combined with *S. aureus*. This study confirmed that the presence of the Gram-positive bacteria did not hinder predation on the Gram-negative bacteria [14]. Correspondingly, in the current study, in the presence of both the Gram-positive test bacteria, PF13 successfully attacked and killed the Gram-negative prey. Additionally, based on the results obtained in the current study, no significant difference in the *P. fluorescens* and *K. pneumoniae* cell count and GC log reductions were recorded for the co-culture experiments versus the dual species assays, which implies that the presence of the Gram-positive bacteria did not significantly hinder predation of PF13 on the Gram-negative prey.

### 3.3. Polymicrobial Assays

To determine whether *B. bacteriovorus* PF13 selectively preys on specific organisms in a polymicrobial community, PF13 was simultaneously cultured with *P. fluorescens*, *K. pneumoniae*, *S. aureus* and *E. faecium*. To monitor the change in concentration of the various prey cells and the predator PF13 in this polymicrobial community, only EMA-qPCR analysis was used. The EMA-qPCR analysis indicated that in the polymicrobial culture, the concentration of PF13 increased by 2.65 logs. For *P. fluorescens*, the GC increased by 0.67 logs (Table 2A). In contrast, for *K. pneumoniae*, the GC decreased by 2.34 logs within 24 h, whereafter the concentration increased (Table 2B). For *S. aureus*, the concentration decreased by 0.53 logs (Table 2C), while for *E. faecium*, the concentration remained relatively constant with 1.74 × 10^7^ GC/mL detected at 0 h and 1.39 × 10^7^ GC/mL detected at 96 h (Table 2D).

The EMA-qPCR results thus confirmed that PF13 preferentially preys on *K. pneumoniae* in the presence of *P. fluorescens*, *S. aureus* and *E. faecium*. As mentioned previously, the exact factors that influence which bacteria are attacked and preyed on or the mechanisms employed by *B. bacteriovorus* to select bacterial prey, have not been identified [1,4,26]. However, although this was beyond the scope of this study, we hypothesise that the unique outer membrane modifications employed by Gram-negative bacteria may make them more or less susceptible to predation by *B. bacteriovorus*. For example, *P. fluorescens* (as well as *P. aeruginosa*) is known to contain a unique outer membrane porin OprF, which has been associated with the adhesion of these bacteria to surfaces, is important for the maintenance of the cell shape and has been found to play an important role in virulence in *Pseudomonas* spp. [27,28,29] Additionally, OprF displays conformational changes in various *Pseudomonas* spp., which leads to cell surface structure alterations which could aid these bacteria in surviving in adverse conditions (such as low-osmolarity) [28,30]. In contrast, *K. pneumoniae* is well-known for its polysaccharide capsule attached to its outer membrane and the subsequent mucoid morphology on standard agar plates [31]. In addition, *K. pneumoniae* has been shown to remodel the lipid A moiety of its lipopolysaccharide layer in order to evade host immune responses and antibiotics such as colistin [32]. In turn, *B. bacteriovorus* has been shown to easily penetrate bacterial capsules and has a unique lipid A moiety in its lipopolysaccharide layer, which may aid the predator in prey cell identification [5,33]. Therefore, it can be hypothesised that in the current study, in the polymicrobial assay, *K. pneumoniae* was preferentially preyed on as it was unable to effectively protect itself from attack by *B. bacteriovorus*, while the outer cell surface of *P. fluorescens* may have been more difficult to penetrate. Varon [11] similarly hypothesised that the structure of the surface of bacteria may render bacteria more or less susceptible to predation, as it has been shown that differences in the cell envelope of different Enterobacteriaceae influenced the attachment efficiency of bdellovibrios. Future studies could thus focus on outer membrane modifications to elucidate prey cell selection by *B. bacteriovorus*.

Rogosky et al. [13] also indicated that predation by *B. bacteriovorus* is not random. To subsequently determine what may drive prey cell selection, the authors examined the prey cells microscopically to determine if prey cell size played a role and they investigated the concentration of plaques produced by *B. bacteriovorus* in combination with the different prey strains [13]. The authors found that the prey cells were all similar in size and that *B. bacteriovorus* produced a similar number of plaques in the presence of all the prey cells [13]. However, while all the prey could support the growth of *B. bacteriovorus*, the predator still preferred certain prey strains [13]. The authors then hypothesised that less “domesticated” strains of bacteria may be preferred by *B. bacteriovorus*; however, the type strain of *S. marcescens* (deemed “domesticated”) was preferred over other bacteria and this result thus did not support their hypothesis [13]. Furthermore, it has been proposed that *B. bacteriovorus* may require an attachment site on the cell wall of the bacteria. However, the specific receptor required by this predator has not been identified [13,34,35], while it has been shown that the production of an S-layer can protect certain bacteria (such as *Aquaspirillum serpens*, *Aquaspirillum sinuosum*, *Aeromonas salmonicida* and *Caulobacter crescentus*) from predation by *B. bacteriovorus* [13,33,36]. Furthermore, chemotaxis may also play a role in predation [13,37,38].

Similar to the Rogosky et al. [13] study, Dashiff et al. [6] investigated dual species assays consisting of two predation sensitive bacteria. The cultures consisted of the following combinations: *Acinetobacter baumannii* (*A. baumannii*) and *K. pneumoniae*; *Enterobacter gergoviae* (*E. gergoviae*) and *K. pneumoniae*; and *A. baumanni* and *Enterobacter cloacae* (*E. cloacae*). However, in contrast to the current study and the Rogosky et al. [13] study, their results indicated that the log reductions obtained in the co-culture assays were comparable to the log reductions obtained for the dual species assays for the respective prey bacteria. The results from the Dashiff et al. [6] study thus indicated that *B. bacteriovorus* does not selectively prey on predation-sensitive bacteria in mixed bacterial cultures. It is thus evident that prey selection may be dependent on the strains *B. bacteriovorus* is cultured with and that prey selection may vary. In the current study, we found that in a polymicrobial culture consisting of two Gram-negative bacteria (which are predation sensitive), and two Gram-positive bacteria, *B. bacteriovorus* PF13 selectively preys on the Gram-negative bacterium, *K. pneumoniae*. This corresponds to the dual species assays where PF13 preferentially preyed on *K. pneumoniae* in the presence of *P. fluorescens*. Future studies could investigate cultures consisting of different combinations of Gram-negative bacteria, which are sensitive to predation by PF13, to determine if this predator always selects certain prey, or whether this predator behaves in the same way as *B. bacteriovorus* 109J (i.e., selective predation dependant on which prey strains are included in the culture experiments). This will advance our understanding of the behaviour of wild-type strains such as *B. bacteriovorus* PF13 and could aid in identifying which combinations of organisms could be targeted using these predators.

It is also important to note that the studies that have investigated the interaction of *B. bacteriovorus* with dual species cultures and complex bacterial communities have employed standard culture-based methods to enumerate the prey and predator bacteria [6,12,13,14]. Although culture-based analysis provides a good indication of whether predatory bacteria degrade other bacteria through the observation of cell count reductions, some bacteria may enter a viable but non-culturable (VBNC) state, which may not be observed with culture-based analysis, but can still influence predator–prey interactions as *B. bacteriovorus* can prey on VBNC cells [13]. Additionally, while investigating the interaction of *B. bacteriovorus* HD100 with a complex microbial community, which consisted of *S. aureus*, *A. baumannii*, *B. cereus* and *K. pneumoniae*, Im et al. [14] highlighted that reductions in cell counts of individual bacterial species could not be accurately calculated using culture-based analysis. The study thus only reported overall cell count reductions and indicated that treatment with *B. bacteriovorus* HD100 resulted in a 68% reduction in the overall pathogen cell count. In the current study, culture-based methods and EMA-qPCR were thus utilised to monitor the concentration of the Gram-negative and Gram-positive bacteria exposed to *B. bacteriovorus* PF13. Ethidium monoazide bromide is an azide-bearing nucleic acid intercalating dye that can enter cells with damaged membranes [39]. The use of this dye thus allows for the exclusion of extracellular DNA, or DNA in cells with compromised membranes (presumed non-viable), from downstream molecular analysis such as qPCR [39]. In combination with qPCR technologies, this dye allows for the quantification of presumably viable cells, including VBNC cells [39]. Furthermore, the use of species or genus specific primers allows for the quantification of specific genera or species within a mixed culture. A previous study by our research group then indicated that the viability and the concentration of *B. bacteriovorus* PF13 and the bacteria co-cultured with *B. bacteriovorus* PF13 could be monitored using EMA-qPCR (detecting both the presumably viable and the VBNC cells) [17]. Therefore, the use of molecular methods such as EMA-qPCR could aid in further elucidating the interactions of *B. bacteriovorus* with different prey strains.

### 3.4. Predation Efficiency

As true predation was confirmed for *K. pneumoniae* and *P. fluorescens* through the formation of plaques on the double-layer agar overlays, the predation efficiency of *B. bacteriovorus* PF13 on only these two Gram-negative prey bacteria was calculated for the co-cultures, the dual species assays and the polymicrobial assay. In addition, the EMA-qPCR results were utilised for the predation efficiency calculations instead of the plate counts, as plate counts were not obtained for the polymicrobial assay, while the GC concentrations were obtained for all the different experimental set-ups. The GC concentrations of *K. pneumoniae* and *P. fluorescens* were subsequently converted to cell equivalents (CFU/mL). *Klebsiella pneumoniae* and *P. fluorescens* has one copy of the *Gyrase A* and *oprl* genes, respectively [40,41]. The cell equivalents were used in the efficiency calculations as outlined by Rogosky et al. [13].

For *P. fluorescens*, 1.5% viable cells remained after 96 h of predation in the co-culture experiment (*P. fluorescens* and PF13) as compared to the control assay (*P. fluorescens* only). In addition, in the dual species assays, only 2.16% and 2.05% of the viable cells remained when *P. fluorescens* was cultured with *S. aureus* and PF13 and *E. faecium* and PF13, respectively, in comparison to the control samples that did not include PF13. In contrast, 70.1% of the viable cells remained when *P. fluorescens* was cultured with *K. pneumoniae* and PF13. Similarly, in the polymicrobial assay, the predation efficiency calculations indicated that relative to the control sample, 48.5% of the *P. fluorescens* cells remained viable.

For *K. pneumoniae*, 1.23% of viable cells remained after 96 h of predation in the co-culture experiment (*K. pneumoniae* and PF13) as compared to the control experiment (*K. pneumoniae* only). In addition, in the dual species assays, only 0.23%, 0.03% and 2.59% of the viable cells remained when *K. pneumoniae* was cultured with *P. fluorescens* and PF13, *S. aureus* and PF13 and *E. faecium* and PF13, respectively, as compared to the control samples (no PF13 present). In the polymicrobial assay, the *K. pneumoniae* cell counts were initially reduced by 2.34 logs after 24 h of exposure to PF13 and the predation efficiency calculations then indicated that at this time point only 0.048% of the viable *K. pneumoniae* cells remained as compared to the control culture at 24 h. However, the *K. pneumoniae* cell counts subsequently increased and the efficiency calculations indicated that after 96 h of exposure to PF13, 1.40% of the *K. pneumoniae* viable cells remained as compared to the control culture.

Overall, our results thus show that in dual species cultures that consist of one Gram-negative and one Gram-positive organism, the presence of the Gram-positive does not negatively affect predation by PF13 on the Gram-negative bacteria, as the predation efficiency was not significantly changed for the dual species cultures versus the co-cultures (for both *K. pneumoniae* and *P. fluorescens*). In contrast, for the dual species assays that consisted of *P. fluorescens* and *K. pneumoniae* exposed to PF13, 0.23% of the viable *K. pneumoniae* remained (which was comparable to the percentages obtained in the co-culture and dual species assays), while 70.1% of presumably viable *P. fluorescens* remained. Similarly, for the polymicrobial assay, 1.4% of the viable *K. pneumoniae* remained, while 48.5% of presumably viable *P. fluorescens* remained. Thus, while PF13 effectively preyed on *P. fluorescens* in the co-culture experiment and in the dual species assays with the two Gram-positive strains, the predation efficiency of PF13 on *P. fluorescens* was reduced in the dual species experiment with *K. pneumoniae* and in the polymicrobial assay. In contrast, the predation efficiency results indicated that, irrespective of the culture conditions and prey strain present, PF13 preferentially preys on *K. pneumoniae.*

Notably, in the polymicrobial assays, the concentration of *P. fluorescens* increased (by 0.67 logs) over the incubation period, and although the concentration of *K. pneumoniae* was reduced after 24 h, from 48 to 96 h, the concentration of *K. pneumoniae* also increased, indicating that regrowth of the bacteria may have occurred. This was in contrast to the results obtained for the co-culture and dual species assays where the concentration of these Gram-negative organisms was reduced and remained low after 96 h of exposure to PF13 (with the exception of the dual species assay consisting of the two predation sensitive strains exposed to PF13 where the concentration of *P. fluorescens* also increased). Thus, the selective predation of PF13 on *K. pneumoniae* may have allowed the *P. fluorescens* cells to proliferate over the 96 h (in both the polymicrobial and dual species assay (*P. fluorescens*, *K. pneumoniae* and PF13)). Hobley et al. [12] hypothesised that proteolytic degradation of prey bacteria in multispecies cultures may result in the cycling of nutrients and this may allow some prey organisms (that would otherwise be reduced) to proliferate in the presence of *B. bacteriovorus*. Additionally, the predation rate on *K. pneumoniae* may have been reduced after 24 h in the polymicrobial culture, as *K. pneumoniae* reached a significantly lower concentration than the other bacteria analysed (<10^3^ CFU/mL vs. >10^6^ CFU/mL). The other bacteria present at a higher abundance after 24 h may thus have acted as decoys which slowed predation on *K. pneumoniae*. Subsequently, we hypothesise that *K. pneumoniae* could utilise the nutrients present in the media (which could have been increased as a result of proteolytic activity) to recover and grow in the presence of PF13.

## 4. Conclusions

As mentioned, prey selection (and predation efficiency on selected prey) may vary based on the predator strains investigated and the origin of both the prey and predator. This was confirmed in the current study where the wild-type *B. bacteriovorus* PF13 strain preferentially preyed on *K. pneumoniae* in the presence of both prey-sensitive (*P. fluorescens*) and non-sensitive (*S. aureus* and *E. faecium*) strains in dual species and polymicrobial cultures. *Klebsiella pneumoniae* is ubiquitous in nature and has been extensively isolated from water sources such as wastewater and it is thus hypothesised that the wild-type *B. bacteriovorus* PF13 may have encountered these types of prey in the wastewater environment it was isolated from, resulting in the significantly high predation efficiencies recorded. Moreover, while a wider range of prey strains in combination with *K. pneumoniae* and PF13 will need to be investigated, this predator could potentially be employed to specifically target this opportunistic pathogen in a mixed bacterial community. For example, PF13 could be employed to target *K. pneumoniae* to remove this opportunistic pathogen from contaminated water sources. Hence, it may be prudent to isolate predatory bacteria from diverse environments such as wastewater, freshwater, marine environments, and soil, or more specifically from the environment they may potentially be applied to and characterise these strains in terms of their prey range and prey preference.

The results obtained also confirmed that *B. bacteriovorus* PF13 does not necessarily prey on Gram-positive organisms, such as *S. aureus* and *E. faecium,* as no plaques were observed. However, as the GC of PF13 increased in the presence of these bacteria, the predator may interact with these bacteria to survive. Future studies should investigate this interaction in more detail (using methods such as electron microscopy to visualise attacked cells and time-lapse fluorescens microscopy to monitor the interaction over time), while the interaction of *B. bacteriovorus* with other Gram-positive bacteria, which may be important in clinical or agricultural settings, should also be investigated to determine which Gram-positive bacteria can be utilised as nutrient or energy sources by these predatory bacteria. It has also been shown that *B. bacteriovorus* secretes lytic enzymes to benefit energetically from Gram-positive bacteria and their biofilms [8]; therefore, the lytic enzymes produced by PF13 should be investigated and characterised. These lytic enzymes could potentially be applied as novel antibacterial, antimicrobial or antifouling agents.

## Figures and Tables

**Table 1:** Cell counts and gene copies (± standard deviation) of *B. bacteriovorus* PF13 in the co-culture, dual species and polymicrobial experiments.

Experimental Group	Prey and Predator Combinations	Culture-Based Analysis			EMA-qPCR Analysis		
		Initial Cell Count (PFU/mL)	Final Cell Count (PFU/mL)	Log Change	Initial Gene Copies (GC/mL)	Final Gene Copies (GC/mL) ^c^	Log Change
Co-culture Experiments	PF13 + *P. fluorescens*	2.10 ± 0.9 × 10^3^	1.30 ± 0.3× 10^9^ (48 h) 2.85 ± 1.38 × 10^4^ (96 h)	+5.79 (*p* < 0.001) +1.13 (*p* = 0.20)	2.38 ± 2.36 × 10^3^	1.39 ± 0.28 × 10^7 ^ (48 h) 9.99 ± 0.89 × 10^6^ (96 h)	+3.77 (*p* = 0.04) +3.62 (*p* = 0.008)
	PF13 + *K. pneumoniae*	1.41 ± 1.22 × 10^4^	2.10 ± 0.17 × 10^9^ (48 h) 5.10 ± 2.77 × 10^3^ (96 h)	+5.17 (*p* = 0.006) −0.44 (*p* = 0.55)	3.86 ± 3.46 × 10^4^	3.89 ± 0.80 × 10^7 ^ (48 h) 2.55 ± 1.01 × 10^7 ^ (96 h)	+3.00 (*p* = 0.04) +2.82 (*p* = 0.13)
	PF13 + *S. aureus*	NVP ^a^	NVP^a^	ND ^b^	2.84 ± 1.90 × 10^3^	5.60 ± 3.40 × 10^4^	+1.25 (*p* = 0.38)
	PF13 + *E. faecium*	NVP ^a^	NVP^a^	ND ^b^	1.73± 1.10 × 10^4^	6.23 ± 4.43 × 10^6^	+2.56 (*p* = 0.296)
Dual species Experiments	PF13 + *P. fluorescens* + *K. pneumoniae*	5.66 ± 1.92 × 10^6^	5.03 ± 0.54 × 10^8^ (24 h) 1.75 ± 0.51 × 10^7^ (96 h)	+1.95 (*p* < 0.001) +0.49 (*p* = 0.004)	2.81 ± 0.014 × 10^6^	3.21 ± 0.98 × 10^7^ (24 h) 7.21 ± 0.037 × 10^7^ (96 h)	+1.06 (*p* = 0.0001) +1.41 (*p* < 0.001)
	PF13 + *P. fluorescens* + *S. aureus*	NVP ^a^	NVP ^a^	ND ^b^	6.31 ± 3.62 × 10^1^	7.09 ± 0.15 × 10^6^	+5.05 (*p* < 0.001)
	PF13 + *P. fluorescens* + *E. faecium*	NVP ^a^	NVP ^a^	ND ^b^	3.24 ± 4.35 × 10^3^	6.37 ± 9.32 × 10^6^	+3.29 (*p* = 0.021)
	PF13 + *K. pneumoniae* + *S. aureus*	NVP ^a^	NVP ^a^	ND ^b^	1.08 ± 0.32 × 10^3^	1.75 ± 0.067 × 10^7^	+4.21 (*p* = 0.0015)
	PF13 + *K. pneumoniae* + *E. faecium*	NVP ^a^	NVP ^a^	ND ^b^	5.03 ± 0.89 × 10^4^	4.29 ± 0.042 × 10^7^	+2.93 (*p* < 0.01)
Polymicrobial Experiments	PF13 + *P. fluorescens* + *K. pneumoniae* + *S. aureus* + *E. faecium*	NVP ^a^	NVP ^a^	ND ^b^	4.18 ± 0.88 × 10^4^	1.86 ± 0.58 × 10^7^	+2.65 (*p* = 0.086)

^a^ NVP—no visible plaques observed on double-layer agar overlays. ^b^ ND—not determined—log changes could not be calculated as no visible plaques were observed. ^c^ Gene copies per mL reported for 96 h unless otherwise indicated.

**Table 2:** Cell counts and gene copies (± standard deviation) of the Gram-negative [*Pseudomonas fluorescens* (A) and *Klebsiella pneumoniae* (B)] and Gram-positive [*Staphylococcus aureus* (C) and *Enterococcus faecium* (D)] bacteria exposed to PF13 in co-culture, dual species and polymicrobial cultures.

Experimental Group	Prey and Predator Combinations	Culture-Based Analysis			EMA-qPCR Analysis		
		Initial Cell Count (CFU/mL)	Final Cell Count (CFU/mL) ^a^	Log Change	Initial Gene Copies (GC/mL)	Final Gene Copies (GC/mL) ^a^	Log Change
**A: *P. fluorescens***							
Co-culture Experiment	*P. fluorescens* + PF13	2.73 ± 1.00 × 10^9^	3.36 ± 3.30 × 10^6^	−2.91 (*p* = 0.11)	1.01 ± 0.0067 × 10^6^	2.67 ± 0.80 × 10^4^	−1.58 (*p* = 0.0001)
Dual species Experiments	*P. fluorescens* + *K. pneumoniae* + PF13	5.17 ± 2.28 × 10^8^	4.98 ± 1.46 × 10^8^	−0.02 (*p* = 0.46)	7.34 ± 4.52 × 10^4^	4.11 ± 0.29 × 10^5^	+0.75 (*p* < 0.0001)
	*P. fluorescens* + *S. aureus* + PF13	3.43 ± 0.37 × 10^9^	3.33 ± 4.71 × 10^6^	−3.01 (*p* < 0.001)	5.76 ± 0.034 × 10^5^	3.53 ± 0.18 × 10^4^	−1.22 (*p* = 0.007)
	*P. fluorescens* + *E. faecium* + PF13	8.53 ± 2.56 × 10^9^	2.20 ± 0.73 × 10^5^	−4.59 (*p* = 0.009)	7.90 ± 0.63 × 10^5^	1.65 ± 0.047× 10^4^	−1.68 (*p* = 0.006)
Polymicrobial Experiment	*P. fluorescens* + *K. pneumoniae* + *S. aureus* + *E. faecium* + PF13	ND ^b^	ND ^b^	ND ^b^	1.15 ± 0.14 × 10^6^	5.34 ± 1.80 × 10^6^	+0.67 (*p* = 0.007)
**B: *K. pneumoniae***							
Co-culture Experiment	*K. pneumoniae* + PF13	6.50 ± 2.17 × 10^8^	1.20 ± 1.07× 10^5^	−3.73 (*p* = 0.095)	2.48 ± 0.93 × 10^7^	3.06 ± 2.79 × 10^5^	−1.91 (*p* = 0.12)
Dual species Experiments	*K. pneumoniae* + *P. fluorescens* + PF13	2.29 ± 2.35 × 10^8^	5.90 ± 2.03 × 10^4^	−3.59 (*p* = 0.038)	8.63 ± 0.92 × 10^6^	2.75 ± 0.51 × 10^5^	−1.50 (*p* = 0.007)
	*K. pneumoniae* + *S. aureus* + PF13	2.67 ± 0.42 × 10^8^	2.00 ± 0.82 × 10^4^	−4.12 (*p* = 0.001)	1.58 ± 0.01 × 10^7^	4.27 ± 0.014× 10^4^	−2.57 (*p* < 0.001)
	*K. pneumoniae* + *E. faecium* + PF13	6.33 ± 3.40 × 10^8^	6.67 ± 2.62 × 10^4^	−3.98 (*p* = 0.057)	1.23 ± 0.008 × 10^7^	4.89 ± 0.078 × 10^5^	−1.40 (*p* < 0.001)
Polymicrobial Experiment	*P. fluorescens* + *K. pneumoniae* + *S. aureus* + *E. faecium* + PF13	ND ^b^	ND ^b^	ND ^b^	1.25 ± 0.96 × 10^6^	5.74 ± 1.04× 10^3^ (24 h) 2.37 ± 7.13× 10^5^ (96 h)	−2.34 (*p* < 0.001) −0.72 (*p* < 0.001)
**C: *S. aureus***							
Co-culture Experiment	*S. aureus* + PF13	1.97 ± 2.59 × 10^9^	2.27 ± 2.50 × 10^7^	−1.94 (*p* = 0.003)	2.86 ± 3.23 × 10^6^	3.76 ± 5.29 × 10^5^	−0.88 (*p* = 0.75)
Dual species Experiments	*S. aureus* + *P. fluorescens* + PF13	4.90 ± 1.14 × 10^9^	1.73 ± 0.13 × 10^8^	−1.45 (*p* = 0.004)	6.49 ± 1.61 × 10^6^	4.84 ± 1.28 × 10^4^	−2.13 (*p* < 0.001)
	*S. aureus* + *K. pneumoniae* + PF13	2.00 ± 0.82 × 10^8^	1.50 ± 0.29 × 10^5^	−3.12 (*p* = 0.026)	2.42 ± 1.62 × 10^5^	2.00 ± 1.59 × 10^5^	−0.083
Polymicrobial Experiment	*P. fluorescens* + *K. pneumoniae* + *S. aureus* + *E. faecium* + PF13	ND ^b^	ND ^b^	ND ^b^	5.23 ± 2.93 × 10^6^	1.55 ± 0.67 × 10^6^	−0.53 (*p* = 0.05)
**D: *E. faecium***							
Co-culture Experiment	*E. faecium* + PF13	1.28 ± 1.67 × 10^9^	4.82 ± 2.62 × 10^7^	−1.43 (*p* = 0.004)	2.64 ± 3.08 × 10^7^	4.35 ± 3.50 × 10^7^	−0.78 (*p* = 0.42)
Dual species Experiments	*E. faecium* + *P. fluorescens* + PF13	1.13 ± 0.11 × 10^9^	5.33 ± 2.62 × 10^6^	−2.32 (*p* < 0.001)	2.37 ± 0.19 × 10^7^	1.07 ± 0.0038× 10^7^	−0.35 (*p* = 0.02)
	*E. faecium* + *K. pneumoniae* + PF13	1.00 ± 0.00 × 10^8^	1.10 ± 0.22 × 10^7^	−0.96 (*p* < 0.001)	1.19 ± 0.025 × 10^7^	3.09 ± 0.021 × 10^6^	−0.59 (*p* < 0.001)
Polymicrobial Experiment	*P. fluorescens* + *K. pneumoniae* + *S. aureus* + *E. faecium* + PF13	ND ^b^	ND ^b^	ND ^b^	1.74 ± 0.026 × 10^7^	1.39 ± 0.16 × 10^7^	−0.098 (*p* = 0.08)

^a^ Final cell count or gene copies after 96 h of incubation with PF13^. b^ ND—not determined—log changes could not be calculated as no visible plaques were observed.

## Data Availability

Data will be made available upon request to the corresponding author: wesaal@sun.ac.za.

## Supplementary Materials

The following supporting information can be downloaded at: https://www.mdpi.com/article/10.3390/microorganisms10040793/s1, Table S1: The primers and cycling parameters utilised for the quantification of *B. bacteriovorus* PF13, *P. fluorescens*, *K. pneumoniae*, *S. aureus* and *E. faecium* with the EMA-qPCR assays; Table S2: Performance characteristics for the qPCR assays to quantify the Gram-negative bacteria, Gram-positive bacteria and the predator B. bacteriovorus PF13; Table S3: Concentration of the Gram-negative and Gram-positive bacteria in the control assays.
