# Supplementary material for: Interaction of Bdellovibrio bacteriovorus with Gram-Negative and Gram-Positive Bacteria in Dual Species and Polymicrobial Communities
Source: Microorganisms. 2022 Apr 9;10(4):793. doi: 10.3390/microorganisms10040793 (PMC9025206; doi:10.3390/microorganisms10040793)
Supplement: Supplementary file 1 [file microorganisms-10-00793-s001.zip › microorganisms-1622513-supplementary.pdf]

Supplementary Information

Interaction of *Bdellovibrio bacteriovorus* with Gram-negative and Gram-positive bacteria in dual species and polymicrobial communities

Table S1 The primers and cycling parameters utilised for the quantification of *B. bacteriovorus* PF13, *P. fluorescens*, *K. pneumoniae*, *S. aureus* and *E. faecium* with the EMA-qPCR assays

Organisms	Primer	Primer Sequences (5′ – 3′)	qPCR Cycling Parameters	Conventional PCR Cycling Parameters	Gene (product size in bp)	Melting Peak (°C)	Reference
***Bdellovibrio* spp.**	Bd347F	GGAGGCAGCAG TAGGGAATA	2 min at 95°C; 50 cycles of 15 s at 95°C and 60 s at 60°C; high resolution melting of 60 s at 95°C, 60 s at 40°C, 1 s at 65°C and 1 s at 97°C	2 min at 95°C; 50 cycles of 15 s at 95°C and 60 s at 60°C; final elongation of 10 min at 72°C	16S rRNA (202)	84.30± 1.00	Van Essche et al., 2009
	Bd549R	GCTAGGATCCCT CGTCTTACC					
***Enterococcus* spp.**	ECST784F	AGAAATTCCAAA CGAACTTG	10 min at 95°C; 50 cycles of 15 s at 95°C and 60 s at 60°C; high resolution melting of 60 s at 95°C, 60 s at 40°C, 1 s at 65°C and 1 s at 97°C	5 min at 95°C; 30 cycles of 30 s at 94°C, 60 s at 59°C and 60 s at 72°C; final elongation of 10 min at 72°C	23S rRNA (75)	79.15 ± 1.00	Frahm and Obst, 2003
	ENC854R	CAGTGCTCTACC TCCATCATT					
***Klebsiella* spp.**	gyrA-A	CGCGTACTATAC GCCATGAACGTA	10 min at 95°C; 50 cycles of 60 s at 94°C, 30 s at 50°C and 30 s at 72°C; high resolution melting of 60 s at 95°C, 60 s at 40°C, 1 s at 65°C and 1 s at 97°C	3 min at 95°C; 35 cycles of 60 s at 94°C, 30 s at 50°C and 30 s at 72°C; final elongation of 10 min at 72°C	*Gyrase A* (383)	87.70 ± 1.00	Brisse and Verhoef, 2001
	gyrA-C	ACCGTTGATCAC TTCGGTCAGG					
***Pseudomonas* spp.**	PS1	ATGAACAACGTT CTGAAATTC	10 min at 95°C; 50 cycles of 30 s at 94°C, 30 s at 58°C and 30 s at 72°C; high resolution melting of 60 s at 95°C, 60 s at 40°C, 1 s at 65°C and 1 s at 97°C	10 min at 95°C; 50 cycles of 30 s at 94°C, 30 s at 58°C and 30 s at 72°C; final elongation of 10 min at 72°C	*oprI* (249)	88.70 ± 1.00	Bergmark et al., 2012
	PS2	CTGCGGCTGGCT TTTTCCAG					
***Staphylococcus* spp.**	PanStaphF	CAATGCCACAAA CTCG	10 min at 95°C; 45 cycles of 30 s at 95°C, 30 s at 61°C and 30 s at	10 min at 95°C; 45 cycles of 30 s at 95°C,	*tuf* (462)	81.50 ± 1.00	Sakai et al., 2004

	PanStaphR	GCTTCAGCGTAG TCTA	72°C; high resolution melting of 60 s at 95°C, 60 s at 40°C, 1 s at 65°C and 1 s at 97°C	30 s at 61°C and 30 s at 72°C; final elongation of 10 min at 72°C	

Table S2 Performance characteristics for the qPCR assays to quantify the Gram-negative bacteria, Gram-positive bacteria and the predator *B. bacteriovorus* PF13

**qPCR Performance Characteristics**	***B. bacteriovorus***	***P. fluorescens***	***K. pneumoniae***	***S. aureus***	***E. faecium***
Efficiency (*E*)	105 ± 1.25	90 ± 1.00	95.25 ± 0.75	96.13 ± 2.33	101% ± 1.00
y-intercept	32.99 ± 2.98	39.72 ± 0.34	33.89 ± 0.285	35.54 ± 1.95	33.15 ± 0.34
Correlation Coefficient (r^2)	0.96 ± 0.07	1.00 ± 0.00	1.00 ± 0.00	0.99 ± 0.00	1.00 ± 0.00
Slope	-2.49 ± 0.46	-3.95 ± 0.057	-3.58 ± 0.04	-3.53 ± 0.14	-3.29 ± 0.045

Table S3 Concentration of the Gram-negative and Gram-positive bacteria in the control assays

Experimental Group	Combinations	Culture-based Analysis			EMA-qPCR Analysis		
		Initial cell count (CFU/mL)	Final cell count (CFU/mL)	Log change	Initial gene copies (GC/mL)	Final gene copies (GC/mL)	Log change
				A: *P. fluorescens*			
Co-culture Experiment	*P. fluorescens* only	2.75×10^9	1.78×10^9	-0.19	5.26×10^5	1.08×10^6	+0.31
Dual species Experiments	*P. fluorescens* + *S. aureus*	3.07×10^9	3.33×10^9	+0.04	1.95×10^6	2.37×10^6	+0.08
	P. fluorescens + *E. faecium*	8.27×10^9	7.33×10^8	-1.05	2.81×10^6	1.20×10^5	-1.37
	P. fluorescens + *K. pneumoniae*	7.40×10^8	2.56×10^9	+0.54	3.01×10^4	2.41×10^5	+0.90
Polymicrobial Experiment	*P. fluorescens* + *K. pneumoniae* + *S. aureus* + *E. faecium*	ND	ND	ND	5.94×10^5	5.58×10^6	+0.97
				B: *K. pneumoniae*			
Co-culture Experiment	*K. pneumoniae* only	5.34×10^8	1.23×10^9	+0.36	1.29×10^7	5.09×10^7	+0.60
Dual species Experiments	*K. pneumoniae* + *S. aureus*	1.67×10^8	1.07×10^7	-1.19	6.83×10^6	5.75×10^7	+0.93
	K. pneumoniae + *E. faecium*	3.00×10^8	6.33×10^8	+0.32	1.05×10^7	2.58×10^6	-0.61
	K. pneumoniae + *P. fluorescens*	4.93×10^8	1.32×10^9	+0.43	4.22×10^6	5.95×10^7	+1.15
Polymicrobial Experiment	*K. pneumoniae* + *P. fluorescens* + *S. aureus* + *E. faecium*	ND	ND	ND	7.67×10^5	1.03×10^7	+1.13

Experimental Group	Combinations	Culture-based Analysis			EMA-qPCR Analysis		
		Initial cell count (CFU/mL)	Final cell count (CFU/mL)	Log change	Initial gene copies (GC/mL)	Final gene copies (GC/mL)	Log change
C: *S. aureus*							
Co-culture Experiment	*S. aureus* only	3.10×10^8	1.37×10^8	-0.36	5.91×10^6	1.17×10^6	-0.70
Dual species Experiments	*S. aureus* + *P. fluorescens*	4.63×10^9	7.63×10^7	-1.78	6.26×10^6	1.07×10^5	-1.77
	S. aureus + *K. pneumoniae*	1.67×10^8	1.07×10^7	-1.19	1.77×10^5	2.90×10^5	+0.21
Polymicrobial Experiment	*S. aureus* + *P. fluorescens* + *K. pneumoniae* + *E. faecium*	ND	ND	ND	4.36×10^6	2.30×10^6	-0.28
D: *E. faecium*							
Co-culture Experiment	*E. faecium* only	1.80×10^9	2.10×10^8	-0.93	2.94×10^7	1.73×10^7	-0.23
Dual species Experiments	*E. faecium* + *P. fluorescens*	2.47×10^9	1.23×10^7	-2.30	1.89×10^7	4.84×10^6	-0.59
	E. faecium + *K. pneumoniae*	1.67×10^8	2.23×10^7	-0.87	1.44×10^7	3.29×10^6	-0.64
Polymicrobial Experiment	*E. faecium* + *P. fluorescens* + K. pneumoniae + *S. aureus*	ND	ND	ND	1.04×10^7	1.28×10^7	+0.09

ND – Not Determined
